# Supplementary material for: Comparison between hydroxyapatite and polycaprolactone in inducing osteogenic differentiation and augmenting maxillary bone regeneration in rats
Source: PeerJ. 2022 May 2;10:e13356. doi: 10.7717/peerj.13356 (PMC9070322; doi:10.7717/peerj.13356)
Supplement: Supplemental Information 1 [file peerj-10-13356-s001.docx]

**Table 1: Immunoreactive score (IRS) assigned for the semi-quantitative immunohistochemical evaluation of the ALP and OCN expression.**

| **A**  **(Percentage of positive cells)** | **B**  **(Intensity of staining)** | **IRS**  **(Multiplication of A and B)** |
| --- | --- | --- |
| 0 = no positive cells | 0 = no colour | 0-1 = negative |
| 1 = <10% of positive cells | 1 = mild | 2-3 = mild |
| 2 = 10-50% positive cells | 2 = moderate | 4-8 = moderate |
| 3 = 51-80% positive cells | 3 = intense | 9-12 = strongly positive |
| 4 = >80% positive cells |  |  |
