# Supplementary material for: Comparison between hydroxyapatite and polycaprolactone in inducing osteogenic differentiation and augmenting maxillary bone regeneration in rats
Source: PeerJ. 2022 May 2;10:e13356. doi: 10.7717/peerj.13356 (PMC9070322; doi:10.7717/peerj.13356)
Supplement: Supplemental Information 2 — One-way ANOVA was conducted to compare MC3T3-E1 cell viability when cultured on scaffolds and 2D culture plates in terms of a number of viable cells (1 × 10^4^ cells). Values were mean difference ± standard deviation. *Asterisks indicate significant differences after a Bonferroni correction for n = 5, at p < 0.05. MTT assays for cell viability were carried out in triplicate. [file peerj-10-13356-s002.docx]

Table 2: Comparison of a number of viable MC3T3-E1 cells when cultured on HA scaffolds, PCL scaffolds, and 2D culture plates. One-way ANOVA was conducted to compare MC3T3-E1 cell viability when cultured on scaffolds and 2D culture plates in terms of a number of viable cells (1x10^4^ cells). Values were mean difference ± standard deviation. *Asterisks indicate significant differences after a Bonferroni correction for n=5, at p < 0.05. MTT assays for cell viability were carried out in triplicate.

| **Days** | **Increment of cells viable number (1x10^4^ cells)** | | |
| --- | --- | --- | --- |
|  | **HA scaffold vs**  **2D culture** | **PCL scaffold vs**  **2D culture** | **HA scaffold vs**  **PCL scaffold** |
| **0** | 2.4342 ± 1.3604* | 1.7198 ± 0.7578 | 0.7144 ± 0.6026 |
| **7** | 11.6118 ± 0.2206* | 4.8429 ± 0.1517* | 6.7689 ± 0.0689* |
| **14** | 0.8877 ± 1.7731 | -4.7788 ± 1.7671* | 5.6665 ± 0.0061* |
| **21** | -4.3367 ± 1.1522* | -7.4276 ± 1.1393* | 3.0909 ± 0.0123 |
